# Advanced 3D Magnetic Scaffolds for Tumor-Related Bone Defects

**DOI:** 10.3390/ijms232416190

**Published:** 2022-12-19

**Authors:** Florina-Daniela Cojocaru, Vera Balan, Liliana Verestiuc

**Affiliations:** Biomedical Sciences Department, Faculty of Medical Bioengineering, Grigore T. Popa University of Medicine and Pharmacy of Iasi, 9-13 Kogalniceanu Street, 700454 Iasi, Romania

**Keywords:** bone tissue engineering, 3D magnetic scaffolds, biomaterials, magnetic nanoparticles

## Abstract

The need for bone substitutes is a major challenge as the incidence of serious bone disorders is massively increasing, mainly attributed to modern world problems, such as obesity, aging of the global population, and cancer incidence. Bone cancer represents one of the most significant causes of bone defects, with reserved prognosis regarding the effectiveness of treatments and survival rate. Modern therapies, such as hyperthermia, immunotherapy, targeted therapy, and magnetic therapy, seem to bring hope for cancer treatment in general, and bone cancer in particular. Mimicking the composition of bone to create advanced scaffolds, such as bone substitutes, proved to be insufficient for successful bone regeneration, and a special attention should be given to control the changes in the bone tissue micro-environment. The magnetic manipulation by an external field can be a promising technique to control this micro-environment, and to sustain the proliferation and differentiation of osteoblasts, promoting the expression of some growth factors, and, finally, accelerating new bone formation. By incorporating stimuli responsive nanocarriers in the scaffold’s architecture, such as magnetic nanoparticles functionalized with bioactive molecules, their behavior can be rigorously controlled under external magnetic driving, and stimulates the bone tissue formation.

## 1. Introduction

In recent years, the bone disorders and diseases has drastically increased worldwide, and this tendency is expected to continue in the near future, due to the risks related to aging of the population, obesity, and cancer incidence [1]. Bone regeneration and healing mechanisms are activated after a bone fracture or lesion, but the restoration mechanism cannot cover large segmental bone defects caused by tissue aging, trauma, bone tumor resection, or infections, and represents a serious problem in orthopedics treatments and the quality of life of the patients [2,3].

Malignant bone tumors can cause large bone defects, resulted from primary bone cancer or from other cancers that have metastasized to the bones. Osteosarcoma and Ewing’s sarcoma are aggressive malignancies that affect the bone and are primary bone cancers typically arising during childhood. Other cancer types, such as breast, prostate and kidney cancers, usually induce severe pains and spinal cord compression, and determine the bone metastases and bone fractures [4]. Due to a lack of curative therapies only 10% of patients with bone metastases survive, as a result of a poor response to treatment [5].

Allograft and autograft bone are traditional treatments for large defects, and their application as fillers in defective bone is based on the osteogenetic characteristics and healing properties, have demonstrated limited success because of specific complications and challenges. Due to osteoinductive and osteoconductive properties, their non-immunogenic characteristics and compatibility with surrounding tissue, autologous bone grafting is still the gold standard for bone tissue replacing [6,7]. Despite all these advantages, there are some disadvantages for autologous grafting, such as the necessity for a new intervention, injury, morbidity in the donor site, risks for inflammation, and bleeding during the surgical procedure [8]. Furthermore, autografts are not recommended for patients with large bone defects because of limited feasibility and availability [9].

Tissue engineering combines biomaterials, cells, and signaling molecules/growth factors in a synergetic way to induce the bone tissues formation [10] and, therefore, tissue engineering strategies are promising alternatives for repairing the critical bone defects generated by tumors resection, trauma, and diseases with skeletal damages. The understanding of biological cellular processes and the interaction of the biomaterials with bone tissues led to the obtaining of various osteogenic 3D biomaterials, from polymers, ceramics, composites, biomimetic architectures for cell growth and extracellular matrix deposition, as artificial or bioartificial scaffolds are able to restore the mechanical and functional properties of the natural tissue [11].

In designing and processing of the scaffolds for bone tissue engineering some requirements have to be considered: (a) the scaffold should provide the mechanical support in the implanted area; (b) must have a porous 3D architecture in order to facilitate the cell migration, vascularization, and bone developing; (c) to be a substrate for bone elements deposition; (d) to be osteoinductive in order to promote osteogenic differentiation; (e) to stimulate the osseointegration by facilitating and enhancing the cellular activity; (f) to be degraded in a controlled manner, in correlation with new bone formation, and to eliminate non-toxic degradation species; (g) to be loaded and controlled deliver drugs or bioactive molecules in order to accelerate the healing process or to limit the side effects; (h) to not cause any inherent or chronic inflammatory response from the body; (i) to be sterilizable without any changes in the scaffold bioactivity [12]. Porosity and pores interconnectivity is a key feature for scaffolds, in order to allow the invasion with cells, the migration of nutrients which assure the cells survival, the proliferation and formation of the extracellular matrix. Generally, the pores must be greater than 100 μm, to assure the migration of osteoblasts and osteoclasts and to develop a new vascularization [13].

Ceramic materials (e.g., hydroxyapatite-HA, dicalcium phosphates-BCP, tricalcium phosphate-TCP and derivatives) have been tested as scaffolds for bone tissue engineering or as inorganic phase materials with osteoconductive properties, but their inherently brittleness reduces the mechanical properties. Therefore, their clinical use as synthetic bone scaffolds is limited [14]. Natural and synthetics polymers, alone or as composites with calcium phosphates (CP), were considered as an improved solution for bone tissue engineering and regeneration. Collagen, hyaluronic acid, and some polysaccharides (chitosan-Cs, cellulose, and their derivatives), or synthetic polymers (poly (lactic acid)-PLA, poly (lactic-co-glycolic acid)-PLGA, poly (ε-caprolactone)-PCL, poly (methyl methacrylate)-PMMA, and so on) combined with ceramics have been investigated as scaffolds with 3D porous architecture [15]. Advanced biomaterials, sensitive to various external or biological stimuli have been also proposed as resources and mediators of de novo tissue formation, and biological compounds (drugs, proteins, or growth factors) or physical parameters (shear stress and electrical forces, magnetic nanoparticles, magnetic field, or UV light) have been included. Due to their stimulating effect on proliferative activity of osteoblasts and rapid tissue regeneration, magnetic scaffolds were manufactured for treatment of bone tissue defects induced by bone tumors, and some magnetic nanoparticles were included for their hyperthermia and/or chemotherapeutics capabilities [16,17].

This review presents an insight of the magnetic scaffolds composition, the methods of fabrication, and their properties, with a special focus on their performances for treatment of bone tissue defects induced by tumors.

## 2. Bone Tissue Organization and Biochemistry

**Figure 1:**
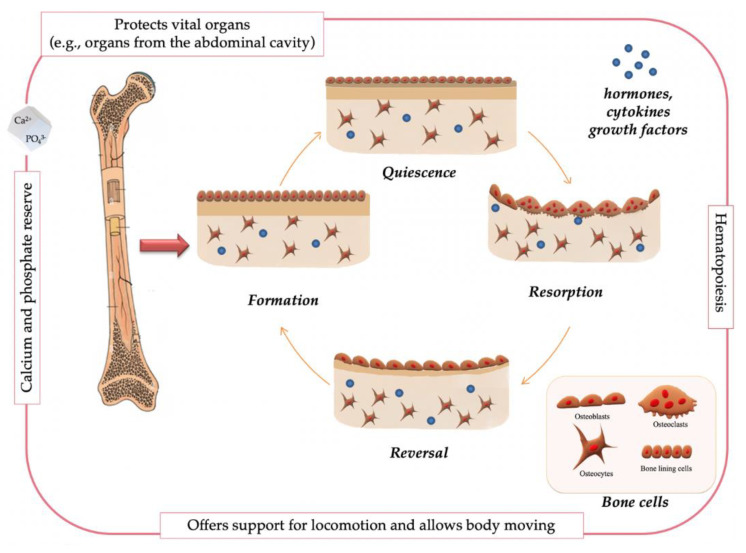
Bone main functions and bone remodeling process.

With a complex inner architecture, bone has a heterogeneous and anisotropic composition, represented by organic and inorganic components, arranged in sophisticated hierarchical levels, from nanoscale to macroscale. Considering the level of the whole organ, the bones can be long or short, flat or tubular. At tissue level, or at macroscale and mesoscopic level, the bone is organized into cortical/compact tissue, represented by Haversian canals and osteons, and trabecular/spongy tissue. The microscopic level of bone is based on an extracellular organic matrix and cells (osteoblasts, osteoclasts, bone lining cells, and osteocytes), while the nanometer level, is based on collagen, mainly type I, various non-collagenous proteins and hydroxyapatite crystals. These complex structures, briefly described above, are dependent by age, tissue site, health conditions, and disorders [18,19]. The hierarchical levels confer to the bone outstanding mechanical features, which make the bone a vital organ because of its support for locomotion and protection of internal organs (brain, bone marrow, and organs from the abdominal cavity). Other bone functions are the maintenance of mineral homeostasis, and calcium and phosphate storage [20,21]. In terms of physiology, bone is a highly dynamic tissue, being constantly remodeled. In normal conditions, there is equilibrium between bone resorption, regulated by osteoclasts and bone formation, coordinated by osteoblasts. At the same time, osteocytes behave as mechanosensors and orchestrators, whereas bone lining cells are believed to connect bone resorption and bone formation. In fact, the four types of bone cells form together a temporary anatomical structure named multicellular unit [22]. Figure 1. summarized in brief, bone remodeling process and the main functions of this key organ.

A complete cycle of bone remodeling can be divided in three main phases: first, osteoclasts set up bone resorption; second phase, entitled reversal, represents the transition between resorption and formation; and, finally, the third phase, bone formation. According to some authors, bone remodeling process also includes an activation phase, in which osteoclast precursor cells are activated, and after formation, follows termination phase, also known as quiescence, when osteoblasts become bone lining cells or differentiate in osteocytes [23]. The perfect equilibrium in bone remodeling is tightly connected with the action of various local and systemic factors: hormones, cytokines, chemokines, and biomechanical stimuli [24]. When formation and resorption phases are disconnected, bone disorders appear, and an imperfect balance generates serious bone disease, such as osteoporosis in case of excessive resorption or osteopetrosis in case of redundant formation [25].

Bone remodeling cycle is regulated by many factors, such as hormones, cytokines, and growth factors [26]. The hormones involved in bone regeneration metabolism can be divided as: growth, gonadal, and calcitropic regulators. The growth regulators hormones are: human growth hormone (hGH), insulin-like growth factor-1 (IGF-1, stimulates formation), glucocorticoids, ghrelin, leptin (dual effect: inhibits resorption and stimulates formation), thyroxin (T3 and T4), adrenocorticotropic hormone (ACTH), and oxytocin; all stimulates formation, but also resorption has a homeostatic effect. Androgens (stimulate formation) and estrogen (dual effect permits formation and inhibits resorption) are gondal regulators hormones, while parathormone (PTH), calcitonin, and vitamin D3 are calcitropic regulator hormones [27].

Supplementary to hormonal regulation, has been established that some growth factors (GFs), such as bone morphogenetic proteins (BMP), transforming growth factor (TGF-β), epidermal growth factors (EGFs), receptor (EGFR), fibroblast growth factors (FGFs), Insulin-like growth factor-1 (IGF-1, the most abundant GF from bone matrix), and WNT and WNT Antagonists, all perform important roles in bone remodeling. Regarding BMPs were identified as five different proteins: BMP-2, BMP-4, BMP-5, BMP-6, and BMP-7, all with substantial osteogenic capacity, known for regulating differentiation of bone marrow mesenchymal cells into adipose tissue, cartilage, and bone. In osteoblast differentiation, TGF-β has opposite roles in comparison with BMPs. From FGFs class, the most important roles have been highlighted for FGF-2, FGF-18, and FGF-23 [28].

Among cytokines, receptor activator of NF-κB (RANK) and receptor activator of NF-κB ligand (RANKL) are the key factors in bone remodeling, since they activate the differentiation of monocyte/macrophage lineage cells into osteoclasts, and together with decoy receptor osteoprotegerin (OPG), are integrated into RANKL/RANK/OPG system, which performs a crucial role in bone resorption. All three are part of the tumor necrosis factor (TNF) superfamily [29].

## 3. Bone Tumors and Tumoral Cells

Generally, based on the lesion’s cellularity compared to the extracellular matrix, nuclear features, the presence of mitotic figures, and necrosis, bone tumors are classified into three grades: low-grade (Grade I), intermediate-grade (Grade II), and high-grade (Grade III) tumors [30]. In the Grade I tumors, the tissues look much like normal bone tissue, and the cells are like normal cells and are less likely to grow and spread quickly. Grade II tumors are more cellular, with a greater degree of nuclear atypia and mitotic activity and nuclear size. Grade III tumors have significant areas of marked variability in the size, shape, and staining of cells and/or their nuclei than Grade II tumors, with occasional giant cells and large necrosis [31]. The bone tumors of high grade are growing very fast, and are the most aggressive group of classic osteoblastic subtype.

Cancer cells developed in bone can have different origin: derived from the bone tissue, bone marrow, or metastasized from a tumor originated elsewhere in the body (breast, prostate, lung, pancreatic, colorectal, etc.) [32]. Malignant primary bone tumors are associated with aggressive growth, and despite surgery and chemotherapy, they are relapse very often. The most common forms of primary bone cancer are osteosarcoma, Ewing’s sarcoma and chondrosarcoma [33]. Cancer cell origin in osteosarcoma is not yet well-defined, but there is evidence favoring the idea of some mutations in mesenchymal stem cells (MSCs) and osteoblast precursors, mostly in the p53 (the protein that regulates osteoblast differentiation, bone formation, and osteoblast-dependent osteoclast differentiation) and retinoblastoma protein (RB) pathway [34]. Rubio et al. have shown that for MSCs deficient in p53 alone or combined with RB, the bone environment performs a vital role in tumor progression [35]. Ewing’s sarcoma, an aggressive tumor, is the most frequently developed in the bones, but can also be found in the extraosseous tissue. Primary bone marrow malignant cells are produced in bone marrow diseases, such as leukemia, lymphoma and multiple myeloma [36]. At the same time, the bone is the most frequent metastatic site for common tumors, such as breast cancer, prostate cancer, and lung cancer [37,38]. The chemokine signaling is hypothesized to play a key role in promoting cancer cell homing to the bone niche [39,40]. Other studies have suggested that integrin binding with bone ECM proteins (osteopontin and bone sialoprotein) may promote cancer cell adhesion to the bone matrix [41,42]. Several reports indicated that bone morphogenetic protein BMP-4 is an important regulator of cells migration and invasion, and an inductor of the epithelial-mesenchymal transition (EMT), that facilitates the cancer cells mobility [43,44]. EMT involves the loosing of intercellular adhesion proteins from the cell surface and epithelial polarization. Due to the destruction of the cell membrane, the dissolution of the extracellular matrix is facilitated by secreting certain proteolytic enzymes, migration into surrounding tissue and invasion in the systemic circulation. Such circulating tumor cells (CTCs) bypass the cell apoptosis due to cell-matrix or cell-cell interactions losing, resulting in activation of phosphatidylinositol 3-kinases/protein kinase B (PI3K-AKT) pro-survival pathways. These tumor cells have also over expressed on surface the integrin associated protein (CD47), involved in protection against to macrophages of the immune system [45].

Once inside the bone, the metastatic cancer cells proliferate, invade, and disrupt normal bone homeostasis through complex and dynamic interactions with the native niche. Bone metastases generally contain a combination of lesion sites that are either osteoblastic (bone forming) or osteolytic (bone resorbing) in nature [46,47]. When cancer cells are present, abnormal bone tissue formation and/or dysregulated bone resorption appears. Some hypotheses indicate that bone breakdown promotes tumor growth through the release of growth factors, such as IGF-1 and TGF-β, by cleaving them from their binding proteins within bone matrix [48,49].

**Figure 2:**
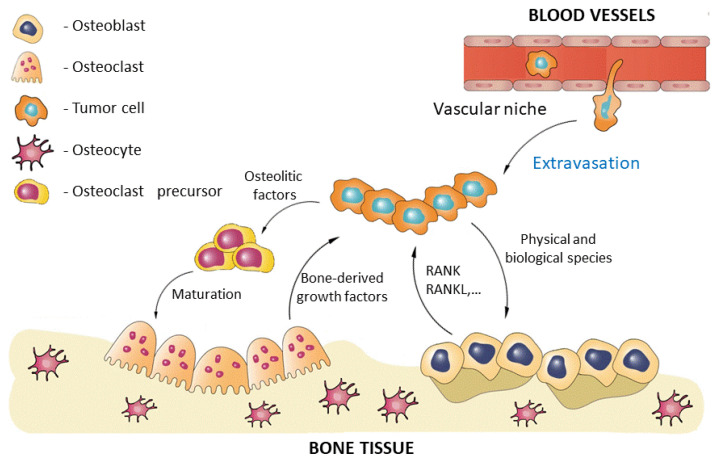
Schematic representation of the cancer cells interactions in bone, including osteoblasts, osteoclasts, and osteoclasts precursors, after activation of the vascular niche.

Cancer cells modify and disrupt both bone and immune systems. Therefore, bone microenvironment and immune system could be involved in promoting tumor physiology and growth. The metastases and tumor growth are attacked by immune system, in particular by T cells, which are destroying the cancer cells [50]. In order to avoid the destruction, the tumor cells need to reduce their immunogenicity and escape the immune recognition. Cancer cells proliferating in the bone are able to influence the microenvironment, by stimulating osteoclasts genesis and bone remodeling (Figure 2). By increasing the bone turnover, the release of different growth factors and cytokines are favored, including the receptor activator of nuclear factor kappa-Β ligand (RANKL). RANKL allows sufficient micro environmental conditions to influence cancer cell migration, attract cancer cells, promote their proliferation, and, finally, the osteoclast-mediated osteolysis is produced [29]. In addition, the T cells are involved in enhanced osteoclasts activity in patients with bone metastases, by releasing the osteoclastogenic factors, including cell signaling cytokine (TNF) and RANKL [51,52]. On the other hand, bone-derived growth factors, such as IGFs and TGFβ (which are released continuously from the bone matrix due to osteoclastic bone destruction) promote cancer cell proliferation and the production of osteoclast-stimulating factors (OSFs). The RANKL expression is affected by OSF in bone marrow stromal cells (BMSCs) and osteoblasts, which interacts with RANK expressed by osteoclast precursor cells (OPCs). Additionally, bone-derived growth factors perform a variety of roles in cancer progression, including cell invasion, angiogenesis, and homing [53].

Hematopoietic stem cell (HSC) niche for bone metastasis considers the microenvironment where HSCs are thought to reside in the bone marrow, and several cell types, such as vascular endothelium and endosteal osteoblasts [54]. Some studies support the hypothesis that bone metastatic cancer cells disseminate in the same manner as HSCs homing in bone marrow, based on several factors that have been implicated in how tumor cells hijack the HSC niche, such as TANK-binding kinase 1-TBK1 (an enzyme with kinase activity, regulates cell proliferation, apoptosis, autophagy, and anti-tumor immunity) and Growth arrest-specific 6-GAS6 (involved in the stimulation of cell proliferation) [55]. Additionally, the bone morphogenic proteins (BMPs) derived from HSCs, such as BMP-2 and BMP-6, are involved in osteoblast differentiation and the osteoblasts maintain the endosteal HSC niche; a positive feedback loop between HSCs and osteoblasts is formed, which leaves an open opportunity for cancer cells to interfere in normal bone formation [56].

Other hypotheses sustain the idea of involving cancer stem cells (CSCs) in bone metastases. Cancer stem cells are cancer cells with tumor-initiating potential, and it is considered to have the capabilities of self-renewal and differentiation [57,58]. In this way, they lead to the development of tumors based on heterogeneous cell populations. As these stem-cell-like properties are required to initiate secondary tumor formation in distant organs, CSCs are expected to play a central role in the development of bone metastases. CD44, a representative marker for stem cells of several cancer types, is an adhesion molecule that binds to the extracellular matrix, mainly hyaluronic acid, and has been implicated in cancer cell migration, invasion, and metastasis [59,60].

To conclude, cancer bone metastasis is a very complex process that involves tumor cells, but also different other types of cells from surrounding tissue and entire body, and cells which are evolved in the metastatic tissue. Some molecular markers can be identified in the early stages of metastasis and used for diagnostic or predictions of evolutions in the case of grade II or III of cancer.

## 4. Bone Tumors Treatment

The treatment of bone tumors is still a challenge, due to the vicious circle between tumor cell proliferation and bone formation phase. The tumor cells will sustain a redundant release of osteoblast RANKL, encouraging osteoclasts differentiation, and activation and intensifying osteolysis process, which will simultaneously release and activate a significant number of growth factors, which will lead to bone destruction and uncontrolled tumoral cells progression [61].

**Figure 3:**
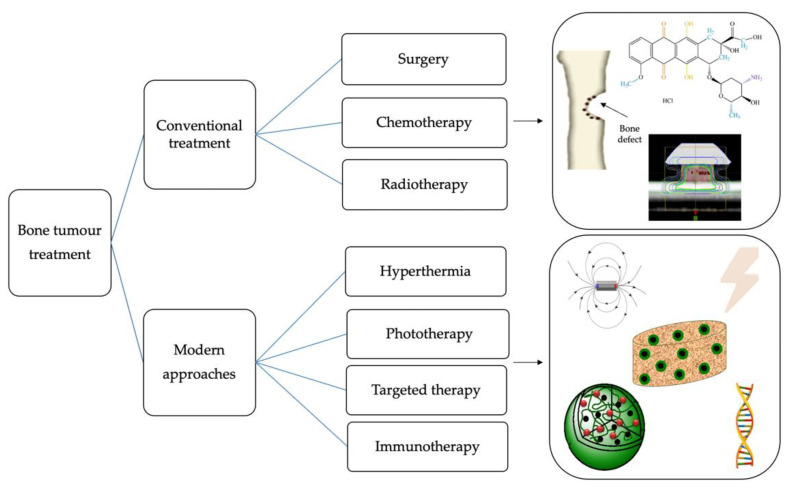
Strategies for bone tumors treatment.

Over the years, the treatment of bone tumors became more complex, including several procedures, such as surgical resection, chemotherapy or radiotherapy, and, recently, alternative therapies, such as hyperthermia, immunotherapy or phototherapy, targeted therapy with functionalized magnetic nanoparticles or stem cells (Figure 3) [62,63].

### 4.1. Conventional Treatment Plans

Osteosarcoma, chondrosarcoma, and chordoma are the most frequent bone tumors, and their treatment plans have been intense studied. Surgery, chemotherapy, and, in some cases, radiotherapy, are the three essential steps included in treatment schemes, which are adjusted considering various aspects, such as the size and site of the tumor, the possibility to surgically resect the tumor, the response to chemotherapy, and the presence of metastases [64,65].

In general, neoadjuvant chemotherapy, based on multiple drugs (methotrexate, adriamycin, cisplatin, ifosfamide, and doxorubicin) with intercalated surgery represents the standard treatment for resectable bone tumors, at children and adults under 40 years old. After induction chemotherapy, surgical resection is a standard in the treatment plan. Radiotherapy is not responsive in case of osteosarcoma, and is therefore not seen as a first-line treatment for resectable tumors, but it is used for surgically unapproachable tumors, or for incompletely resected tumors. For axial skeleton, pelvis, and skull base, modern radiotherapy techniques were reported, such as proton therapy or carbon ion therapy, to overcome the side effects of radiation nearby vital organs [64,65].

The void that results after surgery must be filled with a bone graft material, which should possess the ability to destroy the residual bone tumor cells, by targeted drug delivery, and, at the same time, to sustain the bone defect restoration. This perspective is still at research level, since it has not been yet been put into clinical practice, but it will significantly decrease the side effects of chemo/radiotherapy [66].

### 4.2. Modern Therapies

#### 4.2.1. Hyperthermia

Compared with other therapies, hyperthermia, used alone or as an adjuvant, is easy to perform, satisfactorily kills tumor cells, and increase their sensitivity to chemotherapy, is generally well tolerated and exhibits less complications [67]. For instance, it has been reported that magnetic nanoparticles mixed with hydroxyapatite were able to generate enough heat to kill bone tumor under alternating magnetic field, and when loaded into a scaffold, were effective in photothermal treatment and could entrap active principles involved in stimulation of the bone defects restorations [68]. Furthermore, the clinical trials of Matsumine et al. showed that hyperthermia is as effective as radiotherapy in controlling bone tumor recurrence, after surgery resection [69].

Bigham et al. showed that a 3D bone scaffold loaded with Mg_2_SiO_4_-CoFe_2_O_4_ nanocomposites coated with poly (3-hydroxybutyrate) exhibits a high potential to be used in hyperthermia treatment for bone tumors and bone regeneration [70]. Recently, Zhao et al. developed a superparamagnetic injectable bone cement-based Mn-Zn-Cu-Gd ferrites coated with SiO_2_, able in vitro to self-control hyperthermia around Curie temperature (65 °C) under an alternating magnetic field, with no cytotoxicity and promotion of ability of osteoblasts mineralization [71]. Their strategy was to exploit the advantages of adjusting the Curie temperature (Tc) for an intelligent control of hyperthermia temperature [72,73]. The researchers anticipated that this magnetic bone cement could be considered efficient for improving the thermal safety of hyperthermia in bone tumors treatment [71].

#### 4.2.2. Immunotherapy

Immunotherapy is an alternative and innovative technique, less toxic and non-invasive that exhibit potential to increase the efficiency of treating tumors in the clinic [74]. Activation of therapeutic antitumor immunity is relying on the blockade of immune checkpoints, since tumors are using these pathways as an important mechanism of immune resistance, especially against T cells, specific for tumor antigens [75].

To accomplish a targeted and sustained concentration of imunotherapeutics at tumor sites, scaffolds based on biomaterials with multiple functions have been proposed. In this regard, He et al. combined immunotherapy with a cutting-edge procedure, namely photothermal therapy (PTT), by using a bifunctional bioglass scaffold (BG^@^NbSiR) modified with niobium carbide (Nb_2_C) MXene and loaded with an immune adjuvant (R837). The study concluded that the scaffold, combined with the PD-L1 (programmed death-ligand 1) checkpoint blockade, is able to eradicate primary and metastases tumors in BALB/c mice [76].

Numerous preclinical experiments have encouraged the use of immunotherapies based on tumor vaccines, immune modulators, genetically modified T cells, cytokines, immune checkpoint inhibitors, or combination therapy to osteosarcoma [77,78]. For instance, preclinical murine metastatic models proved to be able to downregulate PD-L1, and upregulate CD80 and CD86 when treated with PD-1 blockade alone, whereas blocking both CTLA4 (inhibitory receptor) and PD-L1 determined a complete control of tumor spread in 50% of animals with osteosarcoma [79,80].

#### 4.2.3. Cell-Based Therapies

Blood transfusions, skin grafts, and the transplantation of bone-marrow or organs can be considered cell therapies given the therapeutic potential of “living cells as specific drugs”. This approach has in center T cells, either naturally occurring, or gene-engineered one, and has been used for lung, prostate, and colon carcinomas [81].

Stem-cell based therapies, a key section of regenerative medicine field, relies on sustaining body repair through stimulation, modulation, and regulation of the endogenous stem cells. In particular, considering the use of stem cells in cancer treatment, this therapy is still at incipient level, since some studies on preclinical models showed contradictory results related to pro-tumor and anti-tumor behavior [82], and raised questions concerning its safety.

Bone tissue engineering (BTE) is a cross-disciplinary branch of regenerative medicine focused on developing bone graft substitutes for the treatment of bone defects obtained after the resection of bone tumors. A great interest on the use of stem cells for bone tumors treatment is paid to design scaffolds for housing of stem cells and signaling molecules. Different types of stem cells, such as human pluripotent stem cells, multipotent stem cells, and progenitor cells are considered in tissue engineering [83].

## 5. Stimuli Responsive Nanocarriers for Bone Tissue Engineering

**Figure 4:**
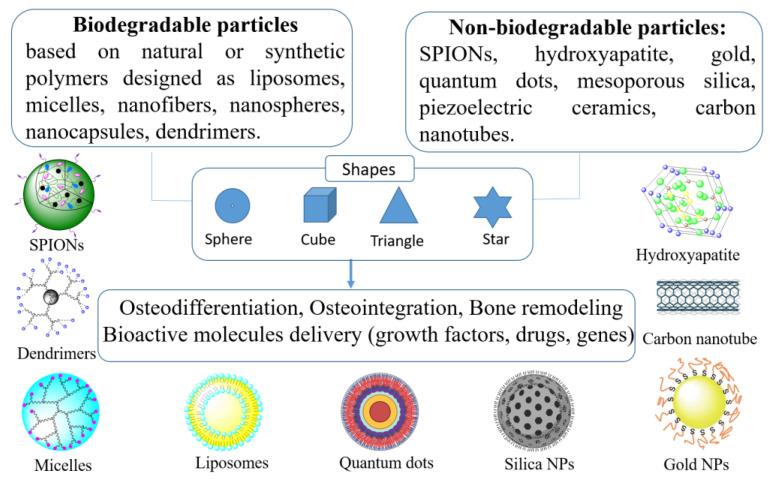
Types of nanomaterials used in bone tissue engineering.

According to a comprehensive review paper, the recent strategies for employing nanotechnology in bone tissue engineering include three different ways: (1) through nanoparticles able to deliver bioactive molecules, growth factors, and genetic material; (2) by nanoparticle-mediated cell labeling and targeting; and (3) by designing nano-based scaffolds [84]. Figure 4 depicts several types of nanomaterials used in bone tissue engineering.

This section is mainly focused on using functionalized magnetic nanocarriers, that accurately mimic the structures occurring in natural bone, and could combine several approaches (for instance, drug delivery and cell labeling) in order to improve the therapeutic efficacy of various bioactive molecules (such as drugs, growth factors, and genetic material), real-time monitoring of tissue regeneration processes, enhance osteointegration, or prevent infections [84,85,86].

These nanoengineered structures quickly respond to a series of stimuli from the surrounding environment (pH, magnetic fields, ultrasounds, and irradiation), serving as stimulus-driven delivery for biologically or chemically active agents [87,88,89]. Their potential also relies on unique features, such as magnetic responsiveness, ability to generate heat, localized magnetic field, improve magnetic resonance (MR) signals, and perform as energy transfer mediators or mechanical force vectors [86].

Generally, these smart nanocarriers are comprised by a magnetic material, a biocompatible surface coating able to ensure stabilization in physiological environmental and multi-functionality, therapeutic agents (drugs, gene, growth factors), and recognition layers (antibodies, receptors, ligands, DNA, and/or oligonucleotides). As magnetic materials, iron oxide nanoparticles (in the form of magnetite and maghemite) have been the most frequently exploited, as they have proved osteoinductive capacity in vitro [90].

The selection of a biocompatible shell is depending on a plentiful key factor, such as (i) biodegradability and biocompatibility, (ii) surface characteristics, functionality, and (iii) the desired size. Therefore, the biocompatible shell could be represented by a variety of materials, such as natural polymers (proteins and polysaccharides- collagen, fibrin, gelatin, alginate, and Cs), and synthetic polymers (poly (L-lactide)-PLLA), or poly (L-lactide-co-glycolic)-PLLGA) [84,86].

Surface conjugation may be achieved through cleavable covalent linkages using the polymers functional groups (amino, hydroxyl, or carboxyl) or by physical interactions, such as electrostatic, hydrophobic/hydrophilic, and affinity interactions [86].

Gene microarray assay and bioinformatics analyses validated that functionalized magnetic nanoparticle (FMNPs) could activate the classic mitogen-activated protein kinase (MAPK) signal pathway [91]. At the molecular level, FMNPs were able to upregulate the long noncoding RNA *INZEB2* and sustain osteogenesis by MSCs [92]. Huang et al. reported that Ferucarbotran suppressed the intracellular H_2_O_2_ through an intrinsic peroxidase-like activity, increased cell cycle progression by the Fe ions released from the lysosomal degradation and modify the expression of the protein regulators [93].

At the same time, functionalized magnetic nanoparticles can be combined with osteogenic medium [94], cyclic magnetomechanical stimulation [95], and BMPs to create an integrated strategy able to promote bone mineralization [96], or can be used as magnetic resonance imaging (MRI) contrast agents to track the implanted cells, scaffold degradation, and bone regeneration.

Recently, it has been reported that growth factors are essential for stimulating the regeneration process [97] because they act as signaling molecules between cells and promote cell maturation and differentiation [86]. Currently, recombinant human BMP-2 and BMP-7 are used in clinical, to enhance bone reconstruction, but there are still some aspects that need to be elucidated, such as the consequent effects on the nature and speed of bone regeneration [98].

Moreover, extracellular stiffness, topography, and remote magnetic actuation are beneficial mechanical signals that can be employed for stem cell differentiation. Magnetic actuation combines the magnetic field with active magnetic nanoparticles [99] capable to target specific cells through mechanosensors. Exposure to a magnetic field could induce modifications of mechanosensors conformation, either by magnetic twisting or clustering, and subsequent activation of biochemical signaling pathways. Generally, targeting could be achieved by the functionalization of a magnetic nanoparticle with ligands that bind to the desired mechanosensor (e.g., integrin recognition motif arginine–glycine–aspartic acid (RGD) peptide, or antibodies to the potassium channel) [100].

In 2016, Ribeiro et al. proposed that magnetoelectric composite materials could connect the magnetic and piezoelectric properties of bone in order to induce a synergistic regenerative effect [101]. Similarly, pulsed and static magnetic fields increased the osteoblast differentiation in vitro, and considerably improved bone repair and regeneration [90,102,103].

## 6. Magnetic Scaffolds for Bone Tissue Engineering

### 6.1. Acting Principle of Magnetic Scaffolds

Although the first attempt to use magnetic fields in medicine was introduced more than 140 years ago, the biological effects of magnetic fields on the body are still intensively studied, for breast cancer therapy, infections, cardiovascular repair, or neural regeneration. Additionally, the studies focused on the nervous and skeletal system [104]. Magnetic fields can be classified in electromagnetic field, pulse electromagnetic fields (PEMFs), static magnetic fields (SMFs) [105], alternating magnetic field (AMFs), or rotating magnetic fields (RMFs), SMFs, and PEMFs being the most used [104].

From biological point of view, as Peng et al. [104] presented in their review, SMFs have different interesting effects, defined by complex molecular mechanisms. For example, using SMFs to control the orientation of kinase domains of epidermal growth factor receptors, resulted in the inhibition of cancer cells proliferation. Regarding the possibility to use magnetic nanoparticles in cancer therapy, most studies are centered on their potential as carriers for bioactive principles, mainly antitumoral drugs, but it was also found that unfunctionalized iron oxide NPs can generate reactive oxygen species, which are known for the disruption of the tumoral cells mitochondrial activity. Moreover, the authors emphasized in their review, that magnetism could influence bone regeneration by activation of specific signaling pathways.

Regarding PEMFs, Bassett figured out that these magnetic fields were helpful in osteonecrosis and orthodontics [106], and magnetic therapy has been considered promising for treatment of many bone diseases, such as osteoporosis, osteoarthritis, spine fusion, distraction osteogenesis, or pseudoarthrosis.

In brief, the mechanism of magnetic therapy is based on the ability of the magnetic field to stimulate the proliferation and differentiation of osteoblasts, promote the expression of BMP and accelerate new bone formation [107]. By incorporation of magnetic nanoparticles decorated with bioactive molecules, such as tissue GFs, into scaffolds and the magnetic moment of the magnetic scaffolds can be continuously controlled, under an external magnetic field and behave, such as a fixed ‘‘station’’, able to regulate tissue formation to the personal needs of the patient [108]. Placed into an external magnetic field, the internal magnetic dipole moment of magnetic nanoparticles is rapidly deflected to the direction of the magnetic field. The attractive magnetic dipole interaction will drive multiple nanoparticles to arrange into ordered magnetic nanochain structures along with magnetic force lines, increasing the magnetic strength [109].

The valuable studies cited at this sub-section, clearly and detailed, reviewed the concept of magnetic field in medicine and less the role of the magnetic scaffold in the bone tissue regenerations in tumor-related bone defects. Magnetic scaffolds, according to their composition (based on bio ceramics, natural biopolymers, and synthetic polymers) and preparation method contribute to defects restoration and bone treatment. Biomimetic magnetic scaffolds are increasingly studied as an innovative bone tissue biomimicry strategy in this field [110].

### 6.2. Preparation Methods

**Figure 5:**
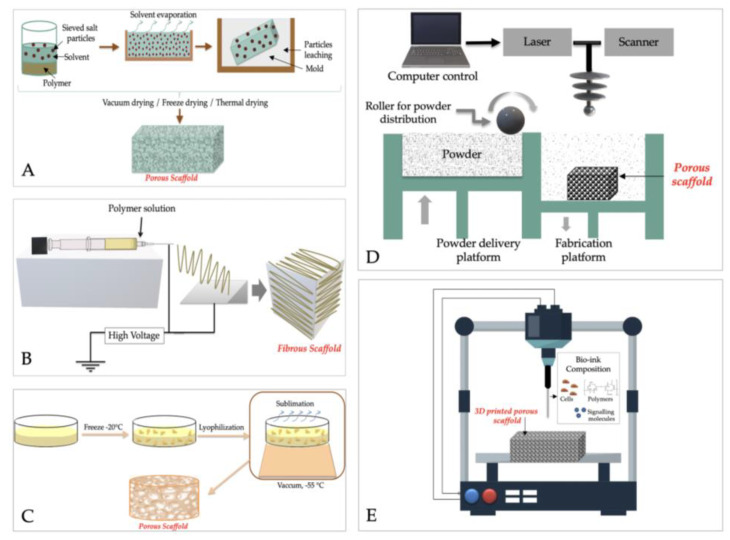
Conventional and modern techniques for designing bone tissue engineered scaffolds. (**A**) Solvent-casting/particulate leaching techniques; (**B**) Lyophilization; (**C**) Electrospinning; (**D**) SLS and SLM; (**E**) 3D Bioprinting.

Currently, there is a wide variety of methods for obtaining bone tissue engineering scaffolds which are used alone or in combination. The “old” ones, known as “conventional methods”, are described by Roseti et al. [111] as “subtractive methods where parts of the material are removed from an initial block to achieve the desired shape”. They mainly include solvent-casting and particulate leaching techniques, gas foaming, melt molding, electrospinning, and lyophilization. Figure 5 presents the basic steps and principles of some of these methods. Unfortunately, it is quite challenging to obtain a complex scaffold with advanced and controlled microarchitecture (pore size, interconnectivity, and shape) using conventional techniques, and, therefore, advanced techniques based on rapid prototyping and computer-aided design were introduced, to strike down these drawbacks. They refer to 3D printing and 3D bioprinting, fused deposition modeling, selective laser sintering and melting, stereolithography, and offer the possibility to design personalized materials with complete control over macro-/microarchitecture, through a precise and repeatable process [112,113].

The main advantages and disadvantages of both conventional and advanced scaffolds fabrication techniques are described in a significant number of reviews [112,113,114]. Recently, Reddy et al. [115] have detailed clearly and concisely the main features of both conventional and advanced scaffolds fabrication techniques. In brief, by solvent casting and particulate leaching can be manufactured highly porous scaffolds with interconnected and controlled pores in a simply and reproducibly manner. but it is time consuming as the solvents can remain in the structure, and the mechanical features of the scaffolds are limited. Similar products, in terms of porosity and mechanical properties, are obtained using melt molding method, but also require time, specific conditions (high temperature in some cases), and are unaffordable. Gas foaming is similar with solvent casting, but even if scaffolds exhibit superior porosity (>90%), the pores are not interconnected (Figure 5).

Lyophilization will generate highly porous scaffolds, but with no control on pores geometry and characteristics, while by electrospinning will be achieved materials with poor mechanical properties, inappropriate for cell seeding [115]. Among advanced techniques, stereolithography was the first developed, as it was based on the use of a photopolymer and ultraviolet radiation. It stands out due to the fact that has the quickest and finest resolution from the 3D printers and the obtained 3D materials have a superior surface texture; however, the materials are brittle, have low impact resistance and strength, and their features are not stable in time [116].

Selective laser sintering (SLS) and selective laser melting (SLM) are two subclasses of powder bed fusion that use a laser to melt and harden a powder polymer-based mixture. Scaffolds with different architectures and controlled porosity can be obtained, but the techniques do not offer the possibility to generate small details, specific boundaries, and sharp corners. Fused deposition modeling (FSM) involves a molten thermoplastic polymer or ceramics, heated above the glass transition temperature threshold, then deposited layer-by-layer. FSM is a promising technique to create custom-made 3D implants but it cannot use natural polymers, and the connectivity and anisotropy of pores cannot be controlled [115,116].

3D-bioprinting uses viable cells encapsulated in the ink, and growth and differentiation factors, and a multitude of shapes and geometric features with specific architecture can be designed. However, its main drawback is given by the fact that post-fabrication steps are necessary to remove the solvents used in the manufacturing process, in some cases proved to be inefficient and it is a technique in the early stages which requires more studies before it can be standardized [115].

Currently, the most advanced method for obtaining scaffolds is 4D-printing, composed of 3D bioprinting and time integrated, as the fourth dimension. In reality, it is used to create intelligent materials with dynamic 3D bioarhitectures, capable to change shape under the action of some stimuli in order to perfectly adapt to the native microenvironments of the defects [117]. In particular, 4D printing is rarely studied for bone scaffolds preparation; only 21 studies were published on Pubmed.gov (key words: 4D printing bone scaffolds), and most of them being focused on 3D printing [118].

### 6.3. State of the Art Regarding Magnetic Scaffolds

The magnetic scaffolds for bone tissue engineering can be divided, according to their composition, based on biopolymers (proteins and polysaccharides), synthetic polymers, and ceramics. Various shapes were considered, from porous scaffolds and hydrogels, to nanofibers and membranes. In this section are summarized representative studies which highlighted the key properties of different types of magnetic scaffolds.

#### 6.3.1. Magnetic Scaffolds Based on Bioceramics

Displaying remarkable features, such as chemical and physical stability, antibacterial and anti-thrombus action, great surface compatibility, mechanical properties, and biocompatibility. Bioceramics have specific biological and physiological functions, and found their usefulness in many biological, biochemical and medical areas. Based on chemically interactions with the living tissue, they can be divided in two main categories: bioinert and bioactive materials [119]. Alumina and zirconia are representative for the bioinert class, being especially used in the manufacturing of joint prostheses, due to their ability to reduce friction and wear, their hardness, and the lack of corrosion in biological medium. However, this class of ceramic materials exhibits some disadvantages, such as inadequate elasticity, high stiffness, and brittleness.

Bioactive glasses (BG) and glass-ceramic, CP (hydroxyapatite-HA, β-tricalcium phosphate- β-TCP, etc.), and diopside (MgCaSi_2_O_6_) are the main classes of bioactive bioceramics. They possess the great ability to sustain in human body the formation of cell and tissue connections [120]. In fact, HA and β-TCP were the components of the first bioceramic bone graft, introduced at the beginning of 1970–1980 decade. Since then, this area evolved considerably. Daculsi et al. listed in an article from 2015, 75 commercially available bioceramic products used in different forms: powders, cements, scaffolds, etc. [121].

Bioceramics bone scaffolds are unique due to their osteoconductivity, osteoinductivity and possibility to be designed as hierarchical structure [122]. Even if the first research about magnetic bioceramic systems was performed relatively recently [107], the results of the studies published since then are good, and the most representative are listed in Table 1. Strictly regarding their application for bone tumors, it is mainly based on their support in hyperthermia treatment. The magnetic nanoparticles will sustain hyperthermia, whereas bioceramics will sustain skeletal reinforcing due to their bioactive features. The challenge is to optimize the composition of magnetic bioceramic scaffolds, in order to prepare a product with good magnetic and bioactive properties, as the magnetic structures with crystalline phase are known for considerable magnetic properties, but, unfortunately, bioactivity is affected by time, due to the weak dissolution rate [123,124].

#### 6.3.2. Magnetic Scaffolds Based on Biopolymers

**Figure 6:**
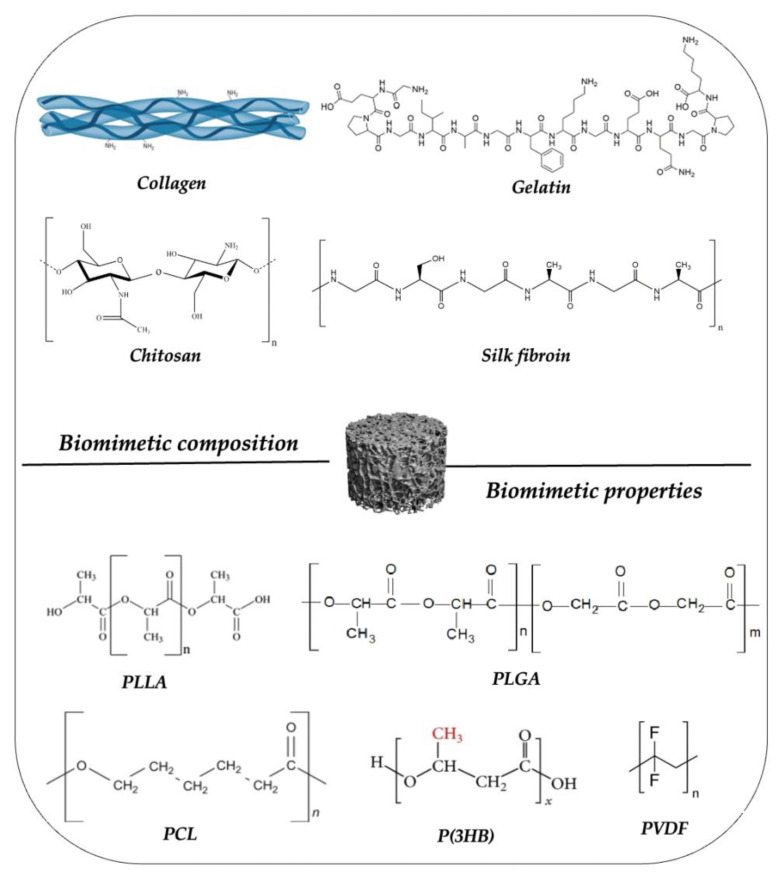
Natural and synthetic polymers used for magnetic scaffolds formulation and preparation.

In recent years, natural polymers are getting wide attention with the perspective of developing high-performance magnetic scaffolds, due to their unique and useful features, such as biocompatibility, biodegradability, and abundant availability. The desired properties can be achieved by blending an appropriate polymer with suitable additives, with good processability and medical performances [142]. A variety of parameters such as chemical composition, degradation kinetics, and mechanical properties of biopolymer composites can be tailored according to the application needs. On the other hand, synthetic polymers have been considered for their homogeneity and excellent mechanical properties. Some classes of polymers used in magnetic scaffolds preparation are presented in Figure 6.

One of the first studies that presented the concept of magnetic scaffolds was published in 2010 by Bock et al. [143] and highlighted two types of magnetic scaffolds. The first one, was based on hydroxyapatite and collagen (70:30 *w*/*w*), crosslinked with 1,4-butanediol diglycidyl ether, formulated as microporous and macroporous lyophilized structures. The second one was obtained from 100% lyophilized collagen in the form of homogeneous porous structures. The magnetization technique consisted in immersing both types of scaffolds, for 15 min in 1 mL ferro-fluid, three different types of ferro-fluids (MNPs with 200 nm size, dispersed in water) were used: FF-DXS (MNPs coated with dextran sulfate and functionalized with groups of sodium sulfate), FF-PAA (MNPs coated with poly-DL-aspartic acid and functionalized with sodium carboxylate), and FF-DP (MNPs coated with starch and functionalized with phosphate groups). The registered magnetization values were considered suitable for the generation of a magnetic gradient in scaffold and in its vicinity. In 2013, Panseri et al. [144] described two different methods for fabricating magnetic scaffolds: method A—based on in situ magnetization, consisted of the nucleation of biomimetic apatite and 7% MNPs, smaller than 50 nm, on self-assembled collagen fibers; and method B—a porous scaffold based on hydroxyapatite and collagen was immersed in the ferro-fluid FF-DP. Scanning electron microscopy data (SEM) proved that the scaffolds prepared using method A showed a distribution of MNPs on collagen fibers, while those prepared by method B exposed an agglomeration of MNPs. Due to promising results have been obtained, the authors continued the study focusing on in vivo [145] and in vitro characterization [146].

##### Gelatin

Gelatin, a derivate of collagen, combined with hydroxyapatite, the main anorganic element from the bone, was frequently used to obtain composite materials, similar to those of bone, intended for bone tissue engineering and regeneration. This protein, also effective for the coating of bio-nanocomposite scaffolds, fabricated using space holder technique from hydroxyapatite powder and magnetite nanoparticles, improving the mechanical and biological features of the scaffolds. The final architecture presents fast response at temperature changes and good potential for hyperthermia applications [147].

Samal et al. prepared gelatin-based magnetic scaffolds, by mixing gelatin solutions with different concentrations of ferro-fluid (magnetite particles, coated with polyacrylic acid, dispersed in water); the mixture was transferred to multilayer polystyrene plates resulting in membranes, further chemically bonded using carbodiimides. The scaffolds showed great hyperthermia ability under exposure to an alternating magnetic field [148].

This protein was also used by Dashnyam et al. to obtain hybrid magnetic scaffolds from gelatin and siloxane by a sol-gel process, including 3% magnetic nanoparticles. Due to the incorporation of MNPs in their structure, a significant improvement of the mechanical properties of the scaffolds was observed [149]. Porous composite bioceramics scaffolds based on gelatin and akeramanite, with inclusion of multi-walled carbon nanotube and magnetic nanoparticles, were evaluated in terms of physico-chemical features (such as morphology, chemical structure, and magnetic and mechanical properties) and in vitro biological behavior (swelling behavior, biodegradation, and biocompatibility using G292 osteoblastic cells) [150].

The association of gelatin with magnetic nanoparticles was also successful for in vivo studies. Gelatin sponges loaded with superparamagnetic iron oxide nanoparticles (SPIONs) were implanted in Sprague-Dawley rats and the results showed that the scaffolds can induce active osteogenesis [151]. Additionally, ultra-small paramagnetic iron oxides (USPIOs) nanoparticles, were incorporated in gelatin-based scaffolds, proposed as valid image-guided and electrically stimulating implant [152].

##### Silk Fibroin

Silk fibroin-SF, a bioactive protein, is a frequent choice in the fabrication of scaffolds for bone regeneration, due to the fact that is structurally homologous to collagen type I, the main protein of the human bone. More precisely, the amorphous spacers between its β-sheets act as nucleation sites in the mineralization process. Other features that recommend SF for bone regeneration applications are represented by its mechanical sturdiness, adequate degradability, bio- and hemo-compatibility [153].

SF was successfully combined with Cs and magnetic nanoparticles, and freeze-casted to achieve scaffolds with controlled porosity for bone tissue engineering applications. Atomic absorption spectroscopy showed that the magnetite nanoparticles were stable in the obtained scaffolds. In vitro, the scaffolds demonstrated an adequate physicochemical activity, regarding the retention of simulated body fluids and biodegradation [154]. Promising results were obtained also, by Tanasa et al., which prepared magnetic scaffolds based on SF and poly (2-hydroxyethyl methacrylate) [155].

Magnetic hydrogels scaffolds were obtained by an electrogelation process of a concentrated solution of SF (8%), with four different types of MNPs (Fe_3_O_4_) incorporated: uncoated MNPs, MNPs coated with human serum albumin (HSA- Fe_3_O_4_), HSA-Fe_3_O_4_ physically conjugated with growth factors basal fibroblasts (HSA- Fe_3_O_4_-bFGF), and without MNPs [156]. Li et al. have also used a concentrated solution of SF (10%) in combination with Fe_3_O_4_ nanoparticles and created core-shell structured magnetic fibers with outstanding magnetic features and adequate in vitro biocompatibility [157]. Three-dimensional porous scaffolds, based on SF and HA, with incorporation of USPIOs, showed osteogenic behavior in vitro and promising results in vivo, after subcutaneously implantation in nude mice, and monitoring by quantitative magnetic resonance imaging [158]. Moreover, Samal et al. emphasized significant hyperthermic properties for magnetic SF scaffolds in the presence of an alternating magnetic field [159].

##### Chitosan

Magnetic biocomposite scaffolds based on Cs and carboxymethylcellulose, a water-soluble cellulose ether, were synthesized by Grumăzescu et al. FeCl_3_ and FeSO_4_ • 7H_2_O have been incorporated in the biopolymers mix solution, with carboxymethylcellulose being used as an ionic crosslinker. The effectiveness of their use as controlled antibiotic delivery systems in vitro, and their interaction with eukaryotic cells, indicated promising perspectives [160]. Heidari et al. presented the fabrication of 3D magnetic composite scaffolds by, in situ synthesis of iron oxide nanoparticles in a 3D composite matrix based on Cs and HA extracted from natural sources. The scaffolds obtained were subjected to a significant number of assays: particle size, TEM, SEM, FTIR, XRD, and TG/DSC. The magnetic properties were studied using a single vibration magnetometer [161].

Fe_3_O_4_, Cs, and poly (vinyl alcohol) (PVA) were the components of magnetic biodegradable nanofibrous membranes obtained by electrospinning. The crystalline and chemical structure of the polymers and iron oxides were not altered by the electrospinning process. The iron oxide NPs were uniformly distributed in the porous membrane. The vibrating-sample magnetometry (VSM assay) showed a weak ferrimagnetic behavior of membranes, but in vitro biocompatibility studies indicated that they can be considered as scaffolds for facilitation of osteogenesis [162].

HA/Cs scaffolds, with incorporation of M-type hexagonal ferrite NPs and lanthanum, included with the aim to control host-to-scaffold immune responses, supported in vitro osteogenic differentiation of rat BMSCs. Scaffold implantation into rat bone defects, followed by histological and micro-CT analysis, revealed their capacity to stimulate new bone formation, which was attributed to the incorporation of NPs and lanthanum [163].

Freeze-dried hybrid Cs/collagen scaffolds with inclusion of nano-HA and Fe_3_O_4_ nanoparticles were prepared by in situ crystallization and evaluated in vitro and in vivo by Zhao et al. [164]. Superior mechanical and structural properties, good in vitro bioactivity, ability to promote cell adhesion and proliferation, and bone regeneration properties after implantation into rat skull defects, were the main features of the scaffolds.

A biomimetic co-precipitation method was used to obtained composite scaffolds based mainly on CsCP, and different concentrations of MNPs (1%, 3% and 5%, respectively); also in addition, three other biopolymers (hyaluronic acid, bovine serum albumin, and gelatin) were separately included in the scaffold’s composition. The scaffold morphology, chemical structure and composition, magnetic features, and in vitro behavior (retention of simulated body fluids, enzymatic degradation, and indirect contact with fibroblasts and preosteoblasts) recommended the scaffolds for bone tissue engineering [165]. In another study, microspheres based on superparamagentic Cs, plasmid, and gelatin proved to be useful tools for angiogenesis in bone tissue engineering, especially in the presence of oscillating and static magnetic fields [166].

In all the studies mentioned above, we have mainly focused on their composition and physico-chemical characterization, and for some of them, the in vitro and in vivo results are presented in Table 2 in order to better highlight the biological features.

#### 6.3.3. Magnetic Scaffolds Based on Synthetic Polymers

In the last 100 years, synthetic materials have been used for the repair and regeneration of various tissues and organs [167]. In vivo, the bone is often subjected to mechanical forces resulting from the actions of muscles and body movements; thus, one of the basic requirements for a scaffold designed to be used in bone tissue engineering is the ability to withstand mechanical stimuli. Considering this key aspect, synthetic polymers have superior mechanical properties, compared to the natural polymers and became attractive candidates for bone regeneration applications [168].

##### Poly-l-Lactic Acid—PLLA

Polylactid acid—PLA—synthesized in different complex ways from its naturally occurring monomer—lactic acid—is a semicrystalline thermoplastic polymer, remarked for its great biocompatibility and biodegradability. PLA, with its stereoisomeric forms: poly (L-lactide) (PLLA), poly (D-lactide) (PDLA), and poly (DL-lactide) (PDLLA), is widely used in various biomedical applications, such as tissue engineering, drug delivery, implants, etc. Regarding bone tissue engineering, PLLA is the most studied PLA form, unfortunately, its low crystallinity leads to inadequate mechanical and thermal properties. As a solution for this drawback, PLLA is used in combination with other polymers or is reinforced with different nanoparticles, resulting materials with good mechanical, thermal, and antimicrobial characteristics [169,170,171].

Interesting scaffolds for bone regeneration have been obtained by combining PLLA with superparamagnetic magnetite nanoparticles, Fe_3_O_4_. Obtained through various preparation techniques (e.g., selective laser sintering or lyophilization), these magnetic scaffolds possess great mechanical properties and non-cytotoxic character, even at high magnetite concentration [172,173]. In other studies, besides PLLA and Fe_3_O_4_, the authors included in the scaffold’s composition and other nanosystems, such as graphene oxide nanosheets [174], or nano-hydroxyapatite (n-HA) [175]. The inclusion of graphene oxide nanosheets enhanced the scaffolds biodegradability and hydrophilicity, while for the scaffolds with n-HA was noted an osteogenic differentiation of BMSCs under a pulsed electromagnetic field.

Magnetostrictive cobalt ferrites (CoFe_2_O_4_) have also been successfully included in formulations of microsphere nanocomposites bioactive scaffolds. In contact with preosteoblasts line MC3T3-E1, the micro-scaffolds improved the cell proliferation rate under dynamic conditions, highlighting their potential to be considered stimulated magnetostrictive biomaterials for bone tissue engineering [176].

##### Poly (Lactic-Co-Glycolic Acid)—PLGA

PLGA, obtained by the polymerization of lactic acid and glycolic acid, is a biocompatible and non-toxic copolymer with a remarkable biodegradability, comparable with the bone formation rate [177]. In combination with hydrophobic Fe_3_O_4_ and processed using electrospininning, magnetic composite scaffolds have resulted. Electron microscopy highlighted a uniform dispersion of the NPs in the polymeric matrix. Compared with PLGA nanofibrous scaffolds, PLGA- Fe_3_O_4_ had a superior behavior in contact with pre-osteoblasts, encouraging cell proliferation and differentiation [178]. Moreover, inclusion of Fe_3_O_4_ in PLGA-HA composite scaffolds led to good results in vitro by improving cell adhesion and proliferation, and in vivo by promoting the repair of radial defect at New Zealand rabbits [179].

Magnetic fibrous scaffolds were also obtained through in situ polymerization of polypyrrole [180] or poly(3,4-ethylenedioxythiophene) [181] on Fe₃O₄/PLGA fibers. In both cases, pre-osteoblasts have been inoculated on the scaffolds under double stimulation (magnetic and electrical), single simulation (magnetic or electrical), or no stimulation, and the best cell growth promoting was obtained for double stimulation [182].

Han et al. [183] used 3D printing for the manufacturing of PLGA scaffolds, which were then coated with engineered iron oxide nanoparticles (γ-Fe_2_O_3_ spherical core capped with a 30 nm-thick polyglucose sorbitol carboxymethylether) through layer-by-layer assembling. The scaffolds coated with NPs showed better results, in vitro (proliferation of rat BMSCs) and in vivo (new bone formed in rat calvarial defects at 8 weeks), compared to those uncoated.

##### Poly (ε-Caprolactone) (PCL)

PCL, biocompatible synthetic polyester, is frequently studied for bone and nerve engineering applications, and for drug delivery systems. Even though this polymer exhibits great features, such as facile processability and reasonable cost, it also has a moderate degradability in simulated body fluids and is hydrophobic, being necessary to be mixed with other polymers, or nanoparticles in order to modulate in vivo activity [184].

One of the first studies dealing with magnetic scaffolds based on PCL was presented by Kim et al. The authors prepared three types of scaffolds with different MNPs (Fe_3_O_4_) concentrations: PCL-MNP5 (5% MNPs), PCL-MNP10 (10% MNPs), and PCL (without MNPs). The incorporation of MNPs in the PCL scaffolds led to an improvement of their hydrophilicity and mechanical properties. PCL-MNPs scaffolds had the ability to support cell adhesion mineralization [185]. Moreover, these types of scaffolds were rigorously studied in vitro [186,187] and in vivo, in terms of biocompatibility and interaction with cells and tissues, and the results indicated a considerable potential of these supports to be used for bone repair and regeneration [188].

Yun et al. prepared magnetic nanofiber scaffolds with two different concentrations of COOH -conjugated MNPs. The study demonstrated, for the first time, that magnetic nanofiber scaffolds sustain the growth, differentiation, and pro-angiogenic property of human dental pulp cells [189]. Similarly, Gloria et al. developed poly (ε-caprolactone) nanocomposite scaffolds, with three different concentrations of MNPs (iron-doped hydroxyapatite—FeHA, with a diameter of 20 nm) [190] and by Alamar Blue test and confocal laser scanning microscopy have shown that the nanocomposite scaffolds can support osteogenic differentiation. The same composition: PCL—FeHA was also used for the preparation of 3D scaffolds obtained by injection/extrusion and deposition of fibers along specific directions [191].

In another study, fused deposition modeling and stereolithography were combined in order to obtain magnetic nanocomposite scaffolds based on PCL and poly(ethylene glycol)—PEG—with adequate morphology and remarkable mechanical properties [192].

Hybrid nanoparticles, based on hydroxylated multi-walled carbon nanotubes functionalized with magnetic iron oxide NPs, were mixed with PCL in order to obtain 3D porous scaffolds, through solvent casting/porogen leaching method. In vitro studies concluded that the presence of carbon nanotubes improved human osteoblast cell line (SAOS-2) attachment to scaffolds, meanwhile the presence of iron oxides encouraged osteoblasts activity [193].

##### Poly (3-Hydroxybutyrate)—P(3HB) and Poly (Vinylidene Fluoride)—PVDF

Polyhydroxyalkanoates (PHAs) is a class of polymers produced by microorganisms, with interesting properties, such as biocompatibility and biodegradability, which make them suitable for tissue engineering applications. It is estimated that exists around 150 types of PHAs, P(3HB) being the most studied and well-characterized [194,195].

Akaraonye et al. [196] highlighted the potential of P(3HB) to be combined with two different forms of Fe_3_O_4_: dried nanoparticles and ferrofluid, for bone tissue engineering applications. The incorporation of uniformly dispersed Fe_3_O_4_ NPs in the polymeric matrix offered superior mechanical properties and crystallinity to the scaffolds, and the capacity to sustain cell (MG-63) attachment and proliferation [196]. Similar results have been observed after including P3HB in the structure of core-shell magnetic nanocomposite; increased compressive strength and sustained cell attachment and proliferation was demonstrated [70].

PVDF is the most researched piezoelectric polymer with an inherent capacity to achieve surface charges under minor mechanical deformations. Piezoelectricity is important in bone tissue engineering applications, since is an inherent feature of natural bone [197].

Magnetostrictive particles of CoFe_2_O_4_ were embedded in the structure of methacrylated gellan Gum/PVDF hydrogel scaffold [198] and PVDF 3D porous scaffold [199], as an encouraging approach for bone regeneration and tissue engineering applications. CoFe_2_O_4_/Methacrylated Gellan Gum/PVDF characteristics, namely porous structure, cell viability higher than 80%, bioresorbability, mechanical, and electrical behavior generated by an applied external magnetic field, were recommended the scaffold for bone regeneration applications [198].

#### 6.3.4. Biomimetic Magnetic Scaffolds for Bone Tissue Engineering

In the late 1950s, biomedical engineer and biophysicist Otto Schmitt introduced the term “biomimetics”, which refers to the transfer of principles from biology to technology, by studying and adapting the manufacturing of artificial processes inspired by natural ones, in order to open new avenues for biomedical issues. Bone tissue engineering is one of the domains that strongly involve the concept of biomimetics, dedicated to developing bone grafts for safe and effective bone repair. A considerable number of scaffolds used in the synthesis stage, the concept of biomineralization, one of the clearest examples of natural process inspiration [200,201].

Creating in laboratory a bone tissue micro-environment, at complex mechanical, electromechanical, and biochemical levels is the next challenge in regenerative medicine of bone. This approach may be achieved by incorporating in the scaffolds structure, growth factors and hormones, as biochemical stimuli. The problems that might arise are related to the control release of this bioactive molecules and their clinical potential. Electrical and/or mechanical external stimuli have been also considered for bone tissue engineering. In fact, application of external mechanical stimuli to implanted scaffolds has been inspired by the process called mechanotransduction [199].

Cells detect the mechanical changes in their microenvironment through a series of structural proteins, such as integrins and actomyosin fibers, and respond through physiological changes, a process known as mechanotransduction [100]. In this process occurred focal adhesion (FA) signaling, actomyosin contraction, stretch activated ion channels, and nuclear associated proteins [202], but of significant relevance are the integrins, heterodimers proteins that transfer forces between inside and outside the cell and cadherins, transmembrane proteins that mediate cell–cell communication. Mechanotransduction can also occur through the nucleus by phosphorylation of emerin [203].

Even if the mechanical forces are the most investigated, electric, and magnetic stimuli were taken into consideration, as novel strategies in bone tissue engineering [199]. Electroactive polymers, such as piezoelectric polymers, e.g., PVDF, can promote preosteoblastic adhesion and differentiation, since natural bone is considered a piezoelectric material [197]. For example, stem cell differentiation can be controlled by stiffness, topographical cues, shear stress, tension, and compression [204,205], cell tension initiating the Rho/ROCK and the mitogen-activated (MAPK) protein kinases pathways of biochemical signaling cascades. Mechanotransduction can be triggered by a reverse piezoelectric effect that delivers nanosinusoidal vibrations called ‘nanokicking to stem cells [206] or by magnetic actuation that uses only the magnetic force alone, and lately, a combination of the magnetic field with responsive magnetic nanoparticles [99].

Biomimetic bone scaffolds, obtained through biomineralization process, mentioned at the beginning of this sub-section, which responded to external stimuli in the attempt to reproduce natural conditions, have been described in Section 6.3.3, showing encouraging results and suggesting that biomimetics can be considered the key for the success of tissue engineered tumor-related bone defects.

## 7. Conclusions and Future Perspectives

Considerable advances have been made towards the preparation of scaffolds for bone tissue engineering. However, the exploitation of new materials and technologies, and the knowledge about bone tissue remodeling represents a new area of research for designing advanced smart platforms intended to restore large bone defects, induced after tumors resection. Magnetic composite scaffolds combine natural or synthetic species with magnetic components able to respond to external stimulation and, consequently, to stimulate the proliferation and differentiation of osteoblasts and ability of osteoblasts for mineralization, to promote the expression of BMP and accelerate new bone formation. Various ceramic biomaterials and versatile polymers are combined with biological molecules (drug, growth factors, signaling molecules, and nutrients) to obtain scaffolds able to support the cells biology and tissue regeneration.

Various magnetic components are tested as stimuli-responsive parts, from magnetite and maghemite nanoplatforms to complex magnetic nanocarriers, that combine several approaches (for instance, magnetic properties and cargo characteristics) in order to improve the therapeutic efficacy, enhance osteointegration or prevent infections.

Combinations of processing methods capitalize the properties of materials with different characteristics, with the aim to create biomimetic systems with predictable response in the living tissue. Polymers and composites imprinting and 3D bioprinting are increasingly considered for bone tissue engineering in order to obtain feedback-regulated architecture. A combination of 3D imprinted/bioprinted technologies and stimuli-sensitive materials allows designing of high biomolecule-loading-capacity scaffolds that respond to external stimuli and modulate the response to the bone microenvironment.

New advanced 4D magnetic scaffolds, integrate time in designing intelligent materials with dynamic architectures, are capable to change shape and microstructure in response to some stimuli, in order to perfectly adapt to the native microenvironments of the bone defects. Progress in fully understanding the tumor cells behavior in bone environment will valorize some biomaterials interactions and specificities for bone components, and will outline new directions in designing advanced magnetic scaffolds for tumor therapy and bone repair.

## Figures and Tables

**Table 1:** Magnetic bioceramics systems.

No.	Composition	Main Properties	Refs.
1.	HA and TCP (65/35) and MNPs synthesized by the group	Promote differentiation and proliferation of osteoblasts in vitro, enhanced the recombinant human BMP-2 (rhBMP-2) expression in vivo	[107]
2.	Magnetized alumina particles (≈350 nm) and platelets (≈5 μm diameter, ≈200 nm thickness) obtained by magnetic freeze casting	Microstructure and hierarchical architecture impact outstanding mechanical properties	[125,126]
3.	Mesoporous bioactive glass (MBG) scaffolds containing iron	Inclusion of Fe induced magnetic features and improved the attachment of bone marrow mesenchymal stem cells (BMSCs) on their structure; sustained hyperthermia and osteoconductivity	[127]
4.	HA combined with different ratios of magnetite: 5%, 10%, and 50%	In vivo, implanted in a serious lesion of the rabbit condyle showed adequate levels of histocompatibility, in the presence of a static magnetic field	[128,129]
5.	Bioactive (Fe^2+^/Fe^3+^)-doped hydroxyapatite (Fe-HA)	Superparamagnetic-like properties, sustained hyperthermia, induced intrinsic magnetization	[130]
6.	Apatite-wollastonite magnetic bioactive glass-ceramic loaded with BMP-2 and hypoxia-inducible factor 1 mutation (HIF1αmu) expressing BMSCs	Sustained bone regeneration and angiogenesis, comparable properties with those of autologous bone graft	[131]
7.	Iron-doped nanocrystalline apatite as a delivery system for doxorubicin	Increased drug release in the presence of low-frequency pulsed electromagnetic field; anti-tumoral effect, as the systems were internalized by cells and doxorubicin was released	[132]
8.	Spherical porous granules of HA containing magnetic nanoparticles	In vitro, killed tumoral cells by generating heat, in the presence of an altering magnetic field	[133]
9.	Fe^3+^-containing hardystonite scaffolds	Bioactive, high specific surface areas, sustained drug delivery	[134]
10.	Diopside (MgCaSi_2_O_6_)-magnetite nanocomposite	Scaffolds with 30wt.% Fe_3_O_4_ showed the highest value of specific absorption rate and increased amount of apatite formed on the surface	[135]
11.	Magnetic 45S5 GB based scaffolds covered with Fe-loaded HA nanoparticles	Biocompatibility, in contact with human osteoblast-like MG-63 cell cultures and mouse bone marrow-derived stroma cell line ST-2; The magnetic coating improved the biological features of 45S5 BG scaffolds	[136]
12.	Multicore-shell magnetic nanoscaffolds were prepared using as core, superparamagnetic maghemite (γ-Fe_2_O_3_), and, as a shell, SiO_2_-CaO bioactive glass	Bioactive heterostructures (sustained HA forming in simulated body fluid, in vitro) and cytocompatible in contact with human mesenchymal cells	[137]
13.	Cs-grafted-PCL nanofibers were incorporated with BGs or magnetic BGs (MBGs), both loaded with Cisplatin	Magnetic BGS loaded with Cisplatin, can be concomitant used for chemotherapy and hyperthermia	[138]
14.	BG Cs porous scaffolds with inclusion of MNPs M-type ferrite (SrFe_12_O_19_)	Scaffolds can be considered for hyperthermia applications, because they acted as photothermal agents and killed residual tumor cells, both in vitro and in vivo	[139]
15.	Mesoporous calcium sillicate/Cs porous scaffolds loaded with M-type ferrite and doxorubicin	The scaffolds showed a strong anti-tumoral ability and sustained bone regeneration, in vitro and in vivo	[140]
16.	Printed β-TCP and of Fe_3_O_4_ nanoparticles/graphene oxide nanocomposite layers	Excellent magnetothermal capacity and excellent bone-forming activity	[141]

**Table 2:** In vitro and in vivo results focused on magnetic scaffolds with biopolymers.

No.	Bio- Polymer	Composition of Scaffolds	In Vitro Results	In Vivo Results	Refs.
1	Collagen	Collagen, Hydroxyapatite/hydroxyapatite functionalized with iron ions (Fe^2+^ and Fe^3+^), different types of magnetic nanoparticles	- in vitro biocompatibility studies have shown that the magnetic scaffolds provided the adhesion and proliferation of mesenchymal stem cells from human bone marrow (hBMSC) - the magnetic scaffolds are cytocompatible and encouraged cells adhesion (human osteosarcoma MG-63) and proliferation, especially in the presence of a static magnetic field	- in vivo studies performed on male rabbits; a permanent magnet was implanted close to scaffolds; the collagen fibers were reorganized - under the effect of the static magnetic field, resulted a highly interconnected trabeculae; bone regeneration was directly correlated to microenvironmental data, mediated by the magnetized collagen fibrils	[143,144,145,146]
2	Gelatin	Gelatin coated nano-scaffolds based on hydroxyapatite and Fe_3_O_4_	- highly biocompatible in contact with fibroblastic cells; showed a positive effect on the cell growth, between 48 h and 72 h	Not performed	[147]
3		Magnetic (water-dispersed poly (acrylic acid) -coated magnetic nanoparticles) gelatin layer scaffold	- multilayered scaffolds with tunable magnetic gradients sustained the attachment of human mesenchymal stem cells (hMSCs) under the presence of an external magnetic field.	Not performed	[148]
4		Gelatin, akeramanite, multi-walled carbon nanotube (MWNT) and magnetic nanoparticles	- in vitro, the scaffolds showed excellent heating performance, indicating their potential to be used in photothermal treatment and offering an adequate temperature for destroying cancer cells; - compared with scaffolds based on gelatin and akeramanite, those containing magnetite and MWNT presented superior viability of G292 osteoblastic cells	Not performed	[150]
5		Gelatin sponges loaded with SPIONs	Not performed	- implanted in the incisor sockets of Sprague-Dawley rats; - at 4 weeks after scaffolds implantation by micro-computed tomography, newly-formed bone was found and a better-preserved alveolar ridge, compared with the group with no implantation	[157]
6	Silk fibroin	SF, Cs, Fe_3_O_4_	- in direct contact with MG-63 osteosarcoma cell line, the scaffolds showed a non-cytotoxic behavior	Not performed	[154]
7		SF, poly(2-hydroxyethyl methacrylate)	- the magnetic scaffolds showed the capacity to support 3T3-E1 preosteoblasts proliferation under the presence of a magnetic field and the fact that a low static magnetic field enhanced in vitro the osteogenic differentiation of cells inside of the obtained scaffolds	Not performed	[155]
8		SF and four different types of Fe_3_O_4_	- cellular cytoskeleton orientated along with the magnetic forces, by applying a magnetic field, which also sustained the proliferation of 3T3-E1 mouse preosteoblasts	Not performed	[156]
9		SF, Hydroxyapatite, Ultrasmall Superparamagnetic Iron Oxide (USPIO)	- in direct contact and seeded with bone marrow-derived mesenchymal stem cells (BMSCs) for 21 days, the scaffold induced an osteogenic differentiation by facilitating the expression of ALP and osteogenic gene	- magnetic scaffolds and magnetic scaffolds seeded with BMSC were bilateral implanted to the back of the subcutaneous tissue of mice; - after 8 weeks of implantation, a growing amount of osteoid deposition and neovascularization was observed for scaffolds loaded with BMSCs, compared with the acellular scaffolds.	[157]
10	Chitosan	Cs, lanthanum hydroxyapatite (LaHA), SrFe_12_O_19_-MLaHA/Cs	- rat bone marrow mesenchymal stem cells (rBMSCs) were used for complex in vitro characterization; - rBMSCs incubated into the MLaHA/Cs, showed a significantly higher viability; - ALP activity of the cells populated on these scaffolds is superior, in comparison with scaffolds based on LaHA and Cs, and scaffolds based on HA and Cs; - osteogenesis-related gene expression levels were also higher for MLaHA/Cs.	- at 12 weeks after implantation in a rat calvarial defect model, MLaHA/Cs demonstrated a superior osteoconductivity, compared with simple scaffolds, - an increased number of collagen fibers were found in the defect areas of the MLaHA/CS and LaHA/CS scaffolds; - new bone formation and osteogenic activity were also better for MLaHA/Cs scaffolds.	[163]

## Data Availability

Not applicable.
